# Using an air purifier as a supplementary protective measure in dental clinics during the coronavirus disease 2019 (COVID-19) pandemic

**DOI:** 10.1017/ice.2020.292

**Published:** 2020-06-11

**Authors:** Bin Zhao, Na An, Chen Chen

**Affiliations:** 1 Department of Building Science, School of Architecture, Tsinghua University, Beijing, China; 2 Beijing Key Laboratory of Indoor Air Quality Evaluation and Control, Tsinghua University, Beijing, China; 3 Department of General Dentistry II, Peking University School and Hospital of Stomatology & National Clinical Research Center for Oral Diseases & National Engineering Laboratory for Digital and Material Technology of Stomatology & Beijing Key Laboratory of Digital Stomatology, Peking University School and Hospital of Stomatology, Beijing, China

*To the Editor*—The current outbreak of coronavirus disease 2019 (COVID-19) continues to spread. The total confirmed cases worldwide were >6.0 million by June 2, 2020, and the number is increasing in countries like the United States and across Europe. Measures including social distancing, border closures, and the epicenter lockdown have been taken to control the spread of this virus. Recently, Dave et al^1^ proposed the challenges of dental care services during the COVID-19 pandemic. We understand that routine dentistry is suspended for precaution of aerosols formed by using drills or ultrasonic devices. Here, we want to highlight the possibility that using air purifiers may serve as a supplementary protective measure in dental clinics and units for organized emergency dental care, especially since the current shortage of personal protective equipment is endangering healthcare workers worldwide.^2^

**Fig. 1.**
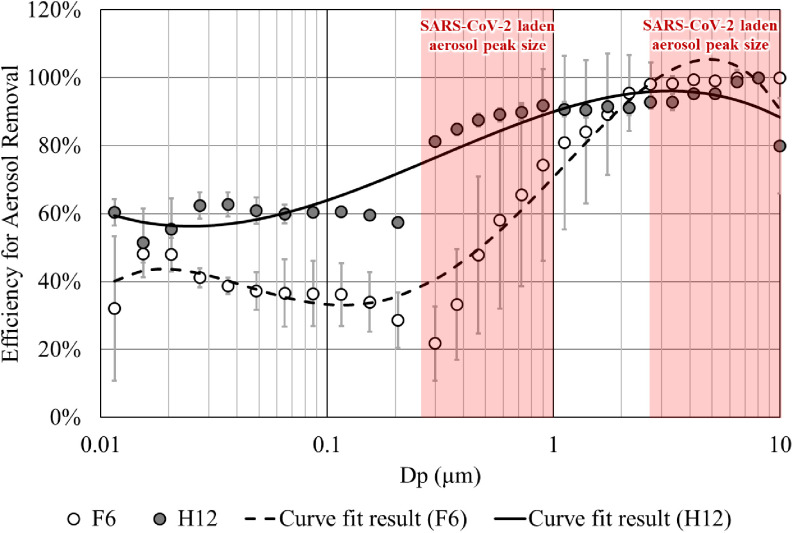
Size-dependent filtration efficiency of fine filters (F6) and HEPA filters (H12).

During dental treatments, saliva may become aerosolized, and microorganisms in such aerosols from the oral cavity contribute to the spread of infectious diseases.^3^ Our previous study proved that running air purifiers in suitable locations can remove aerosols in dental clinics significantly, resulting in the reduction of dental healthcare worker (DHCWs) exposure to aerosols ranging from 80% to 95%.^4^ Regarding to severe acute respiratory syndrome coronavirus 2 (SARS-CoV-2), a recent study found that the virus appears most commonly in aerosols in the submicron (0.25–1.0 μm) and supermicron ranges (>2.5 μm).^5^ Hence, we measured size-dependent filtration efficiency of air purifiers integrated with 2 types of widely used filter media respectively: fine filters (F6 class) and high-efficiency particulate air filters (HEPA, H12 class) (Fig. 1). On average, an air purifier with F6 class filter media removed 54% of aerosols that may carry airborne SARS-CoV-2, while the one with H12 class filter media removed 83% of such aerosols. Therefore, for dental treatments generating a large amount of aerosols, air purifiers with HEPA may be more effective and protective for DHCWs than air purifiers with fine filters. Also, air purifiers with F6 class filter media may still have some efficacy. Notably, the measured filtration efficiency was obtained by measuring aerosol concentration before and after air flow through the filter media on first use. For actual air purifier use, the air is recirculated through the filter media, resulting in a higher efficiency of aerosol removal in enclosed spaces, including dental clinics and units.

Further studies may be needed to provide more direct evidence for the protective effectiveness of air purifiers in dental clinics and units. However, considering the urgent need for emergency dental care during the COVID-19 pandemic, it is highly advisable to use air purifiers as an easy-to-use, portable, inexpensive, and high-efficiency precaution measure, especially in situations where air purifiers are already available.
